# What causes treatment failure - the patient, primary care, secondary care or inadequate interaction in the health services?

**DOI:** 10.1186/1472-6963-11-111

**Published:** 2011-05-20

**Authors:** Per G Farup, Ivar Blix, Sigurd Førre, Gjermund Johnsen, Ove Lange, Rune Johannessen, Hermod Petersen

**Affiliations:** 1 Dept. of Research, Innlandet Hospital Trust, Gjøvik, Norway; 2 Dept. of Cancer Research and Molecular Medicine, Norwegian University of Science and Technology, Trondheim, Norway; 3 Dept. of Medicine, Nordmøre and Romsdal Trust, Kristiansund, Norway; 4 Saupstad Health Centre, Trondheim, Norway; 5 Dept. of Surgery, St. Olavs Hospital, Trondheim, Norway; 6 Dept. of Medicine, Nordmøre and Romsdal Trust, Molde, Norway; 7 Dept. of Public Health and General Practice, Faculty of Medicine, Norwegian University of Science and Technology, Trondheim, Norway

## Abstract

**Background:**

Optimal treatment gives complete relief of symptoms of many disorders. But even if such treatment is available, some patients have persisting complaints. One disorder, from which the patients should achieve complete relief of symptoms with medical or surgical treatment, is gastroesophageal reflux disease (GERD). Despite the fact that such treatment is cheap, safe and easily available; some patients have persistent complaints after contact with the health services. This study evaluates the causes of treatment failure.

**Methods:**

Twelve patients with GERD and persistent complaints had a semi-structured interview which focused on the patients' evaluation of treatment failure. The interviews were taped, transcribed and evaluated by 18 physicians, (six general practitioners, six gastroenterologists and six gastrointestinal surgeons) who completed a questionnaire for each patient. The questionnaires were scored, and the relative responsibility for the failure was attributed to the patient, primary care, secondary care and interaction in the health services.

**Results:**

Failing interaction in the health services was the most important cause of treatment failure, followed by failure in primary care, secondary care and the patient himself; the relative responsibilities were 35%, 28%, 27% and 10% respectively. There was satisfactory agreement about the causes between doctors with different specialities, but significant inter-individual differences between the doctors. The causes of the failures differed between the patients.

**Conclusions:**

Treatment failure is a complex problem. Inadequate interaction in the health services seems to be important. Improved communication between parts of the health services and with the patients are areas of improvement.

## Background

Optimal treatment gives complete relief of many disorders. But even if such treatment is easily available, cheap and safe, some patients have persisting complaints despite contact with the health care system. One such disorder, from which the patients should achieve complete relief of symptoms with medical or surgical treatment, is gastroesophageal reflux disease (GERD). Patients with GERD followed up in meticulously performed clinical trials achieve nearly without exception symptomatic relief and normalized quality of life [1]. In contrast, experience from daily practice and pragmatic studies shows that a substantial proportion of the patients have significant and persisting complaints and reduced quality of life despite treatment in primary and secondary care [2-5]. Overall, this is a significant problem for the health care and the patients since the prevalence of potentially curable disorders is high, e.g the prevalence of GERD is 10-20% [6]. This study evaluates the causes of treatment failure. Is it due to the patient, primary care, secondary care or inadequate interaction in the health services?

## Methods

### Patients

This study was part of a follow-up study of patients with GERD in Norway [5]. The diagnosis was based on typical symptoms and endoscopic findings of esophagitis. Patients with moderate or severe symptoms as judged from a questionnaire were asked to participate.

### Design

One of the authors (HP) interviewed the patients. The interview was semi structured with an interview guide with open questions which focused on the patients' wellbeing, and their satisfaction and experience with treatment, contact with primary and secondary health care, and their opinion of reasons for the treatment failure. The interviews were taped, transcribed and evaluated by the authors who agreed upon a group of patients with persistent, significant symptoms typical of GERD.

Then 18 doctors, six general practitioners, six gastroenterologists and six gastrointestinal surgeons (the gastroenterologists and surgeons were working in hospitals) evaluated and interpreted the transcribed interviews and completed a questionnaire for each of the patients. The doctors were selected based on their position in the health care system, interest in and experience with GERD, and willingness. They had no interaction with the selected patients.

In Norway, all patients have to contact a general practitioner in primary care who has a "gatekeeper" function. If necessary, the general practitioner refers the patient to secondary care (the hospital or out patient clinics) for further evaluation and examinations.

### Questionnaire

The questionnaire completed by the doctors consisted of 31 questions regarding causes of treatment failure. Additional file 1 shows the questionnaire. The questionnaire was constructed by consensus among the authors after several meetings, but it has not been formally validated. The questions were divided into four groups; causes related to the patient, primary care, secondary care and interaction in the health services. The questions were answered with yes, no or n.a. and express the doctor's impression of the patient's opinion. Six questions dealt with the patient, e.g. "Has the patient avoided to contact the doctor?" and "Has the patient been afraid of new investigations such as gastroscopy?". Eight questions dealt with primary care, e.g. "Has the patient had a feeling of being refused or misunderstood by the primary care?" and "Has primary care given incorrect information about side effects of drugs?" Twelve questions dealt with secondary care, e.g. "Has the patient had a feeling of being refused or misunderstood by the secondary care?" and "Has deficient information from secondary care about potent drugs and higher dose contributed to insufficient treatment?". Five questions dealt with interaction in the health care system, e.g. "Could improved communication between primary and secondary care have helped the patient?" and "Could general agreement between specialities about indication for surgical treatment have helped the patient?".

The proportion of "yes" in each group of questions was calculated, i.e. three out of six "yes" answers (50%) concerning the patient had the same value as four out of eight "yes" answers (50%) concerning primary care. Because the total number of "yes" answers varied between the doctors, the scores for each doctor were adjusted to at total score of 100% divided into the four reasons for treatment failure.

### Statistics

Descriptive statistics and ANOVA have been used for presentation of the results and comparisons between the groups respectively, and p < 0.05 has been judged as statistically significant.

### Ethics

The project was recommended by Regional Committees for Medical and Health Research Ethics (REK), Trondheim, and Norwegian Social Science Data Services (NSD), Bergen, Norway. Written informed consent was obtained from all participants before enrolment.

## Results

**Figure 1:**
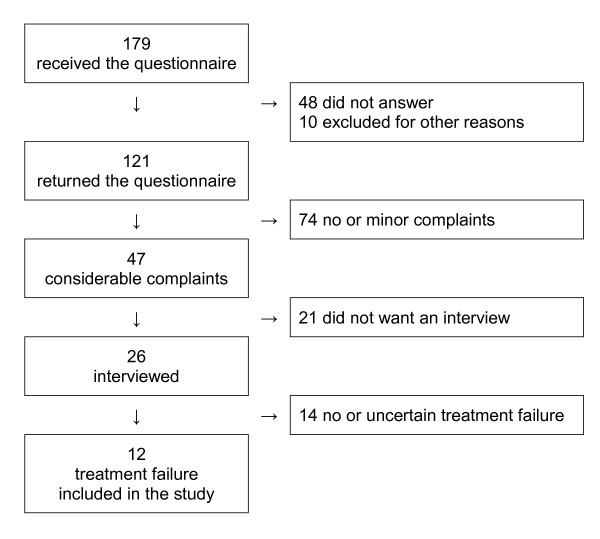
**Selection of patients for the study**.

Twelve out of 179 patients in the follow-up study were included in this study. Figure 1 shows the selection of participants in detail. Some patients with significant complaints were excluded because symptoms were present only when treatment was stopped or other disorders like irritable bowel syndrome were misinterpreted by the patient as GERD. Table 1 gives the patients' characteristics. The mean age of the 18 doctors who evaluated the interviews was 52 years (range 39-64 years), six were specialists in general practice (three men and three women), six gastroenterologists (all were men) and six gastrointestinal surgeons (five men and one woman).

**Table 1:** Patient characteristics.

Variables	Number/mean	Range
Number	12	
Men/women (no)	9/3	
Age (years)	49	30 - 72
BMI (Body Mass Index) (kg/m^2^)	28	24 - 33
Duration of GERD (years)	20	8 - 48
Hiatal hernia (no)	9	

The results are given as number (no) or mean with range

**Figure 2:**
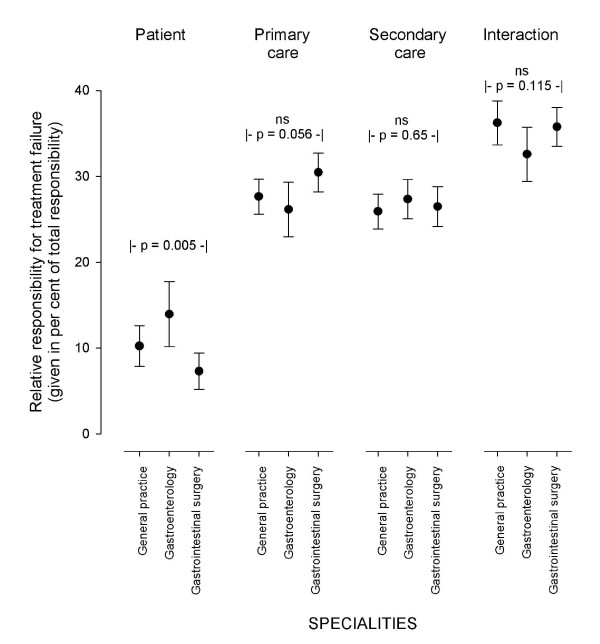
**The responsibility for treatment failure divided between the patients themselves, primary care, secondary care and interaction in the health services as evaluated by specialists in general practice, gastroenterology and gastrointestinal surgery**. The results are given as mean with 95% CI of the mean.

**Figure 3:**
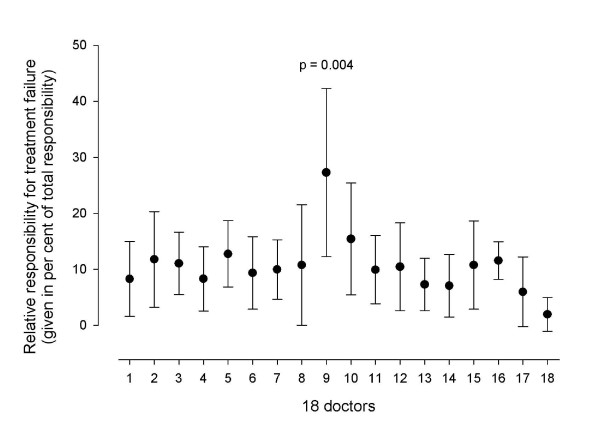
**The figure shows the individual doctors' evaluations of the patients' relative responsibility for treatment failure**. The results are given as mean with 95% CI of the mean. The differences between the doctors were statistically significant (p = 0.004).

Inadequate interaction was the main cause of treatment failure, followed by failing primary care, secondary care and the patient him/her self; the relative responsibilities for treatment failure were 35%, 28%, 27% and 10% respectively. Figure 2 shows how doctors with different specialities evaluated the causes of treatment failure. Except for some disagreement about the patients' responsibility, there was no significant disagreement between the groups. There was, however, significant disagreement (p = 0.004) between the individual doctors' evaluation of the patients' responsibility (Figure 3). They also disagreed about the responsibility of the primary care (p = 0.009, data not shown), secondary care (p = 0.001, data not shown) and inadequate interaction (p < 0.001, data not shown).

**Figure 4:**
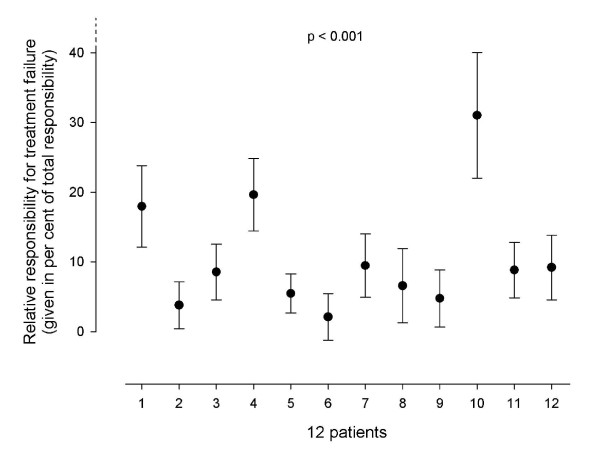
**The figure shows the individual patient's relative responsibility for treatment failure as judged by the group of doctors**. The results are given as mean with 95% CI of the mean. The differences between the patients were statistically significant (p < 0.001)

Figure 4 shows the doctors' evaluation of the individual patient's responsibility for treatment failure. The patients' own responsibility varied significantly (p < 0.001).

## Discussion

The study aimed at describing how patients with persisting complaints despite contact with the health care system assessed the cause of treatment failure. The patients' presentations of their experience with the health care system were independently evaluated and interpreted by doctors with different specialities and background. Although the intention was to have the patients' own opinion about the health care system, the doctors' interpretations of the patients' statements probably have influenced the results which therefore represent a combined patient/doctor evaluation and not only the patients' own perceptions. Particularly concerning the patients' own responsibility and interaction in the health care system, the results express in large the opinion of the doctors after interpretation of the patients' statements.

Treatment failure occurred in all parts of the health care system. Faulty interaction/communication between primary and secondary care and between specialists in gastroenterology and gastrointestinal surgery appears, however, to be the most important factor. The patients told that general practitioners had expressed lack of information from secondary care and uncertainty about the responsibility, and that there was disagreement upon surgical treatment. Similar communication problems are well known internationally. General practitioners lack information from the hospital concerning investigations, treatment and follow-up plans, and the referral letters from primary care to hospitals are inadequate [7,8]. In a Norwegian study, lack of information between primary and secondary care was judged as a health hazard for elderly patients [9]. GERD might represent a special challenge for good communication. A study from USA, where, however, the health care organization differs from that in Norway, showed that 29% of all consultations for GERD resulted in referral from one part to another in the health service [10].

The criticism against primary care was insufficient information about the disease (causes, complications and prognosis) and treatment alternatives (high dose drug treatment and surgical interventions). Some patients felt refused or misunderstood. In addition to the same criticisms, secondary care was blamed for inaccurate diagnostics and lack of referral to other specialists. It is known that these patients have uncovered needs for health services [2,3]. The problem seems to be the communication between the patient and the doctor. The doctor does not grasp the symptom severity, sleep disorders, eating problems, high use of over the counter drugs and reduced quality of life, and does not perceive the patient's incorrect understanding of causes, treatment and prognosis [2,11,12]. Highly effective health services with focus on number of procedures (gastroscopies) at the expense of communication with and clinical follow-up of the patients could in part explain the problems. Routine use of validated patient-reported outcome instruments (e.g. disease specific quality of life questionnaires) and easily available guidelines (algorithms) have been proposed to improve the doctors' perception of the patients' overall situation and avoid over- and under treatment [13,14].

The patients themselves could not discharge from responsibility. Some had shrunken from consulting and resigned themselves to the complaints, some feared side effects of drugs and omitted drug treatment, and some were afraid of investigations (e.g. gastroscopy) and surgery.

The cause of treatment failure varied between the patients. Some patients had to take the main responsibility themselves, whereas the health services were totally responsible for patients who had severe complaints despite following all recommendations.

In large, groups of doctors with heterogeneous background (specialists in general practice, gastroenterology and gastrointestinal surgery) had concurrent evaluations, except for a minor difference concerning the patients' responsibility. It is gratifying that the groups of doctors did not tend to blame each other. There were, however, rather huge differences between the individual doctors' evaluations. The individual discrepancies are perhaps as expected, differences of opinions between doctors are well known from other topics, but the agreement between the groups was a pleasant finding.

This study gives no information about the prevalence of failing treatment because the total number of patients treated for GERD in the study period is unknown, a large proportion did not answer the written inquiry or was unwilling to participate, and patients with mild or intermittent symptoms were excluded. Other studies indicate that 2/3 of the patients have some symptoms and that 3-5% have severe symptoms and reduced quality of life [3,4]. Neither does this study show how to avoid treatment failure. But the study clearly shows that something has to be done and hints at initiatives to improve the communication with the patient and within the health services.

### Strengths and limitations

A combination of qualitative and quantitative methods was used. The interviews were performed with a qualitative method and evaluated and interpreted by the doctors who scored a questionnaire. Strengths and limitations of such a method are unknown. Normally, interviews are analysed by one person or a small group who achieve consensus. In this study, the interviews were evaluated and scored by a group of independent doctors unaware of each others' evaluations. This is likely to be a strength since any bias related to one person's evaluation or consensus in a small group, is omitted. The validity is supported by the agreement between the groups of doctors, and the disagreement between individual doctors makes it likely that individual evaluations of interviews are prone to be biased. The method was chosen because we thought it might give more valid results than traditional qualitative methods do.

The interviews were semi structured. An evaluation of the recordings and transcriptions could indicate that the structure was too loose and that the interviewer might to some extent have influenced the patients' opinions.

The questionnaire filled in by the doctors was not formally validated. It is therefore some uncertainty regarding the completeness of the questionnaire and possible overlap of the questions.

Nearly half of the patients with considerable complaints were unwilling to an interview. Although unknown, this selection has probably had insignificant effect on the external validity of the study.

## Conclusions

Treatment failure is not a simple problem easily eliminated with one intervention, it is a complex problem. Even for the individual patient there is not one single cause. Faulty interaction and communication in the health services was in this study the most important factor, followed by insufficient handling of the patients in primary and secondary care. Sometimes the patients themselves are partly responsible. The agreement of these conclusions between groups of doctors with different specialities was high and gratifying, whereas the disagreement between individual doctors was large and disquieting. The study shows that something should be done to reduce treatment failure, and hints at improved interaction and communication in the health services and with the patient (e.g. validated patient-reported outcome instruments and easily available guidelines) as valuable interventions.

## Competing interests

At the time when the follow-up study of patients with GERD was performed and the interviews were made (in 2003), all authors had in one way or the other been sponsored by AstraZeneca or WyethLederle, two pharmaceutical companies producing proton pump inhibitors used for the treatment of GERD. The authors have no competing interest the last five years.

## Authors' contributions

HP has been responsible for the follow-up study and the design of the qualitative part of this study, and has performed all the patient interviews. He has actively participated in the design of the quantitative part of the study and in writing the manuscript. PGF is the main responsible for the design and accomplishment of quantitative part of the study, the data analyses and for drafting and writing the manuscript. PGF, IB, SF, GJ and OL have together with a group of external doctors read, evaluated and scored the transcribed interviews. All authors have participated in the planning of the study, selected patients for the study based on the interviews, given valuable comments to the manuscript and approved the final version.

## Pre-publication history

The pre-publication history for this paper can be accessed here:

http://www.biomedcentral.com/1472-6963/11/111/prepub

## Supplementary Material

Additional file 1 **The questionnaire filled in by the doctors**. The doctors filled in one questionnaire for each patient based on the transcribed interview of the patient. Click here for file
