# Supplementary material for: What causes treatment failure - the patient, primary care, secondary care or inadequate interaction in the health services?
Source: BMC Health Serv Res. 2011 May 20;11:111. doi: 10.1186/1472-6963-11-111 (PMC3126699; doi:10.1186/1472-6963-11-111)
Supplement: Additional file 1 — The questionnaire filled in by the doctors. The doctors filled in one questionnaire for each patient based on the transcribed interview of the patient. [file 1472-6963-11-111-S1.DOC]

**Questionnaire for evaluation of the interview of the patient**

| **Questions** | **Yes** | **No** | **Not known** |
| --- | --- | --- | --- |
| **Causes related to the patient** |  |  |  |
| Has the patient refused to consult the health care system? |  |  |  |
| Does the patient accept his/her complaints/symptoms and therefore doesn’t want treatment? |  |  |  |
| Has the patient taken less drugs than prescribed? |  |  |  |
| Is the patient afraid of a new gastroscopy and therefore has persistent complaints? |  |  |  |
| Is the patient afraid of a (new) 24 hour pH-measurement or other supplementary examinations and therefore has persistent complaints? |  |  |  |
| Is the patient afraid of an operation and therefore has persistent complaints? |  |  |  |
| **Causes related to primary care** |  |  |  |
| Has the patient had the impression of being refused or misunderstood by primary care? |  |  |  |
| Has insufficient information about GERD from primary care physicians resulted in suboptimal treatment? |  |  |  |
| Has inadequate information from the primary care about potent drugs or use of high doses resulted in suboptimal treatment? |  |  |  |
| Has primary care given incorrect information about the possibility of drug-related adverse events? |  |  |  |
| Has primary care given incorrect or insufficient information about surgical treatment? |  |  |  |
| Has the patient understood the information given by primary care? |  |  |  |
| Has lack of referral of the patient from primary care to secondary care contributed to insufficient treatment? |  |  |  |
| Could a regular follow-up of the patient in primary care have given satisfactory effect of the treatment? |  |  |  |

| **Causes related to secondary care** |  |  |  |
| --- | --- | --- | --- |
| Has the patient had the impression of being refused or misunderstood by secondary care? |  |  |  |
| Has the diagnostic procedures (gastroscopy, 24 h pH-monitoring, motility examinations etc.) been insufficient in secondary care? |  |  |  |
| Has insufficient information about GERD in secondary care resulted in suboptimal treatment? |  |  |  |
| Has inadequate information from the secondary care about potent drugs or use of high doses resulted in suboptimal treatment? |  |  |  |
| Has secondary care given incorrect information about the possibility of drug-related adverse events? |  |  |  |
| Has secondary care given incorrect or insufficient information about surgical treatment? |  |  |  |
| Has the patient understood the information given by secondary care? |  |  |  |
| Has lack of referral of the patient from one specialist to the other in secondary care contributed to insufficient treatment? |  |  |  |
| Has primary care received insufficient information from secondary care? |  |  |  |
| Has the patient received insufficient information from the secondary care concerning follow-up by primary care? |  |  |  |
| Could a regular follow-up of the patient in secondary care have given satisfactory effect of the treatment? |  |  |  |
| Has the follow-up of this patient in secondary care been inadequate (i.e. should secondary care have understood that the patient was in need of regular follow-up)? |  |  |  |

| **Causes related to interaction in the health care system.** |  |  |  |
| --- | --- | --- | --- |
| Could better communication between primary and secondary care have helped the patient? |  |  |  |
| Could better communication between the gastroenterologist and the surgeon have helped the patient? |  |  |  |
| Could a well written patient information about the disease when the diagnoses was verified have helped the patient? |  |  |  |
| Could a general clarification of the responsibility of primary care and secondary care have helped the patient? |  |  |  |
| Could a general agreement about the indications for surgical treatment among doctors with different background have helped the patient? |  |  |  |
